# Assessing the Linkages between Knowledge and Use of Veterinary Antibiotics by Pig Farmers in Rural China

**DOI:** 10.3390/ijerph15061126

**Published:** 2018-05-31

**Authors:** Xiujuan Chen, Linhai Wu, Xuyan Xie

**Affiliations:** Institute for Food Safety Risk Management, School of Business, Jiangnan University, Wuxi 214122, China; xjchen@jiangnan.edu.cn (X.C.); xiexuyan1@yahoo.com (X.X.)

**Keywords:** farmer, veterinary antibiotics use, knowledge, behavior probability model, China

## Abstract

Improper use of veterinary antibiotics (VAs) has led to antibiotic resistance and food safety issues that are harmful for sustainable development and public health. In this study, farmers’ knowledge influencing their usage of veterinary antibiotics was analyzed based on a survey of 654 pig farmers in Funing County, China. A behavior probability model was constructed, and a Matlab simulation was used to evaluate the dynamic changes in farmers’ behavioral choice regarding VAs use. The survey results showed that the 654 pig farmers’ knowledge of VAs were relatively poor, along with a high occurrence of improper behavior. Specifically, 68.35% of the 654 surveyed pig farmers admitted their violation of VAs use regulations, while 55.50% among them overused and 24.31% among them misused VAs. The simulation results showed that the probability of improper VA use decreased with the increase in farmers’ knowledge about VA use specification, and when farmers’ knowledge about the hazards of VA residues increased. However, when farmers had a high level of knowledge about relevant laws and their penalties, there was still a high probability of improper VA use.

## 1. Introduction

Antibiotics are the most important finding in the 20th century for controlling bacterial infections and protecting health [1]. In addition to human treatment, antibiotics have been widely used in agriculture, the food industry, and aquaculture [2]. Veterinary antibiotics (VAs) are widely used in the treatment and prophylaxis of diseases in food-producing animals and in non-therapeutic applications [3,4]. However, the misuse or overuse of VAs is the culprit for increasing antibiotic resistance and food chain contamination [5,6]. Improper use of VAs, on one hand, leads to a high proportion of VA residues that pollute the ecological environment and exacerbate antibiotic resistance. On the other hand, VA residues may accumulate in animals and enter the food chain in the form of chemical hazards, thus causing food safety risks that endanger the health of consumers (i.e., public health) [7,8,9]. It is noteworthy that the development of antibiotic resistance has exacerbated the overuse of antibiotics in veterinary drugs [10], while the release of antibiotics into the environment has accelerated the development of antibiotic-resistant bacteria. This results in a vicious cycle that poses a tremendous threat to the ecological environment and public health.

Since antibiotic resistance has become a common global problem, [11,12], there is increasing concern regarding VAs in developing countries. China is not only the largest producer and user of antibiotics in the world [1,13], but also the largest pig producer and consumer [14]. In 2013, antibiotic consumption in animals accounted for approximately 52% of the total antibiotic consumption of approximately 162,000 tons in China [15]. The negative effects of improper use of VAs by pig farmers are evident to varying degrees in the vast rural areas of China [16]. In many countries around the world, including China, there are a large number of small-scale farmers of meat-producing animals who are the direct users of VAs. In China, farmers tend to overuse VAs, use human antibiotics, or do not follow the withdrawal time recommendations due to their poor knowledge of VAs and the pursuit of economic benefits from meat-producing animals [14].

Minimizing antibiotic resistance should be the responsibility of all members of society [17]. The crucial role of farmers in shaping and preserving multifunctional agro-ecosystems, has been highlighted by agricultural scientists over the past decades [18]. It has been pointed out in some studies that improper antibiotic use by farmers is closely related to their knowledge of antibiotics [19,20]. Kuipers et al. (2016) [21] found that professionally trained farmers (i.e., farmers with higher knowledge level) tend to use less VAs in dairy herds. However, the possible use of VAs by farmers with different knowledge levels and under different regulatory policies (e.g., in China) has been rarely reported. Therefore, this study empirically investigated the knowledge and use of VAs by pig farmers in rural areas in China. A behavior probability model was constructed based on the knowledge of pig farmers that affected their VA use. The dynamic changes in farmers’ behavioral choice regarding the use of veterinary antibiotics, was then observed by Matlab simulation, when considering their knowledge regarding VAs and the different government regulation environments. Based on the findings, policy recommendations were made to regulate improper VA use by farmers.

## 2. Materials and Methods

### 2.1. Sample Site

**Figure 1:**
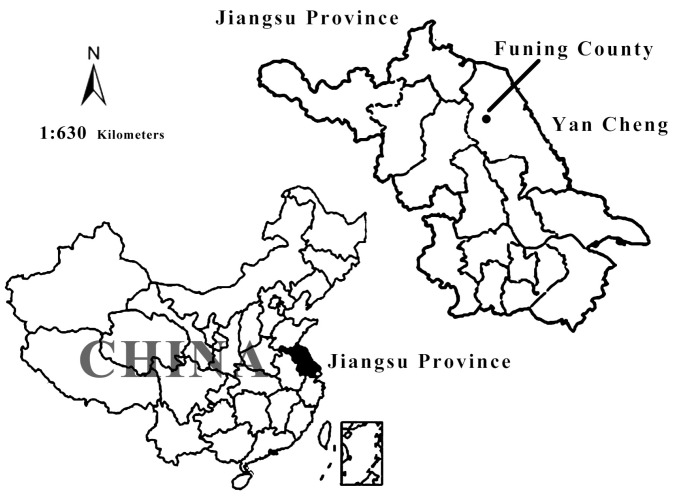
Location of the survey area in China. Note: This is merely a schematic diagram and does not cover the issue of territorial sovereignty.

Funing County (located in Jiangsu Province) was selected as it is a famous pig farming base in China (Figure 1), known as “the hometown of piglets”. Pig farming is an important source of family income for farmers in Funing, and more than 50% of the pigs were produced by small-scale household farming. As small-scale household pig farming where VAs are directly used by farmers will persist over a long period of time in China, investigating the use of VAs in Funing has important practical significance.

### 2.2. Study Design

Prior to the formal survey, a preliminary survey was conducted among pig farmers in Xinlian Village, Sanzao Town, Wangji Village, Longwo Village, and Shuanglian Village in Funing County. A final questionnaire was developed after problems were identified and solved based on the findings of the preliminary survey. The formal survey was conducted by random sampling and home visits in all 13 towns/villages in Funing County. Since face-to-face interviews can effectively avoid the respondents’ possible misunderstanding of survey questions and improve the response rate, the survey was performed by properly trained investigators (postgraduate and doctoral students) who were familiar with the questionnaire and interview process. A total of 654 valid questionnaires were collected for the final analysis.

### 2.3. Instruments

The questionnaire was developed based on the literature review and the authors’ field observations [14,16,22]. The questionnaire was divided into three parts. The first part was designed to collect the demographic characteristics of respondents including gender, age, education, annual production, farming income, and years of farming experience. In the second part, the use and knowledge of VAs were assessed. In view of the diversity and complexity of improper use of VAs, the three most common types: overdose (addition of VAs at a higher than specified concentration), use of human antibiotics instead of VAs, and non-compliance with withdrawal time requirements were investigated. To assess the respondents’ knowledge about VAs, their knowledge about VA use specifications, hazards of VA residues, and relevant laws and their penalties were examined. Theirs level of knowledge was scored on five-point Likert scale where 1 = no knowledge, 2 = little knowledge, 3 = moderate knowledge, 4 = good knowledge, and 5 = complete knowledge. The third part examined the effect of government regulation the use of VAs by farmers. The respondents were asked about the frequency of spot checks of pig farmers’ VA use by local government regulators, how and to what extent government spot checks affected pig farmers’ VA use, whether farmers were punished for improper use of VAs according to law, and what was the effect of punishment. Note that, strictly in China’s newest Regulations on Administration of Veterinary Drugs, there is no permitted use of human drugs on animals

## 3. Model Approach and Simulation Scenarios

This paper referred to a literature for the modeling and scenario methods [22].

### 3.1. Model Construction for Farmers’ Behavior Choice

#### 3.1.1. Basic Model Assumptions

Due to the fact that farmers operate in a complex environment, the simulation could not take into account all the factors that may affect their VA use. Therefore, this study focused on how the differences in farmers’ knowledge affected their VA use during pig farming. The following assumptions were made:

(1) There are only two choices—either proper or improper—for pig farmers regarding the use of VAs. Proper use refers to the use of VAs in a correct and reasonable way according to requirements. Improper use comprises of one or more behavior of VA overdose, use of human antibiotics, and non-compliance with withdrawal time requirements.
(2) Pig farmers are economically rational. Their use of VAs follows the cost–benefit approach.
(3) The government makes spot checks of farmers’ VA use during pig farming. Farmers will be subject to financial penalties, pressure of public opinion, and moral pressure, if improper use is discovered.
(4) Pig farmers’ choice regarding VA use is a dynamic process affected by the behaviors of peers in real-world situations.

#### 3.1.2. Farmers’ Knowledge

In the simulation experiments, pig farmers were the primary actor in economic activity. Their knowledge and cognitive capacity were the main factors affecting their estimation of expected return [23], thus influencing their use of VAs. According to the literature research and the author’s field observations, the farmers’ knowledge was summarized into three categories: knowledge of VA use specification, knowledge of hazards of VA residues, and knowledge of relevant laws and their penalties, represented by *φ_i_*_1_, *φ_i_*_2_, and *φ_i_*_3_, respectively. As the five-point Likert scale was used in the measurement of the farmers’ knowledge level, it was assumed that *φ_i_*_1_, *φ_i_*_2_, and *φ_i_*_3_ take a value in [1,5], respectively, where 1 means no knowledge and 5 means complete knowledge. Since the knowledge level of each farmer is not exactly the same in reality, the values were given for *φ_i_*_1_, *φ_i_*_2_, and *φ_i_*_3_, respectively, in the simulation.

#### 3.1.3. Farmers’ Expected Returns

Based on the above basic assumptions, the farmers’ expected returns were related to government regulation in their behavioral decision regarding the use of VAs. To regulate the use of VAs, government regulators make spot checks to monitor pig farmers’ VA use, and punish the improper use of VAs in accordance with laws and regulations. Government regulation and punishment of pig farmers for improper VA use have an impact on their use of VAs. Therefore, the farmers’ expected returns can be described as follows.

Farmers’ expected return from proper VA use is:

(1) $$W_{1}=G$$

Farmers’ expected return from improper VA use is:

(2) $$W_{2}=(1-q)\times (\Delta G+G)+q\times (\Delta G+G-C_{1}-C_{2})$$

where ΔG=θ×G, where *G* is the farmers’ return from proper VA use; Δ*G* is the farmers’ extra return from improper VA use; *C*_1_ are the financial penalties imposed by government regulators on farmers for improper VA use; *C*_2_ are the social costs of discovered improper VA use for farmers including pressure from public opinion and moral pressure, etc.; *θ* is the ratio of farmers’ increased return from improper VA use to that from proper use; *q* is the probability of the farmers’ improper VA use to be checked by government regulators.

#### 3.1.4. Behavior Probability Model

As pig farmers’ VA use is affected by multiple factors, the choice probability for VA use varies among farmers. Sun et al. [23] developed a mathematical model of behavior probabilities to assess the probability of choosing a certain behavior under the general reward expectation on that behavior. For individual pig farmers, behavior probability is a description of behavioral uncertainty, that is, the probability of a farmer choosing a certain use of VAs in the “behavior set”. Correspondingly, for the pig farmer group, behavior probability is the proportion of individual farmers who choose a certain use of VAs in the group. If all individuals in the group have the same return expectation on each use of VAs, they will all choose the same use of VAs, and there is no need to discuss behavior probability. However, in fact, there is a big difference in farmers’ return expectation on each use of VAs. The differences in cognitive capacity and bias regarding VA use specification, hazards of VA residues, and relevant laws and regulations among each individual actor in the group lead to different probabilities for each farmer in choosing the use of VAs. Based on the literature [23] and the knowledge of farmers, a behavior probability model was developed in this study to simulate the farmers’ VA use during pig farming under different return expectations.

According to the assumptions, the farmers’ VA use was simplified into two categories: either proper use *a*_+_ or improper use *a*_−_. The behavior set was *A* = {*a*_+_, *a*_−_}. The following behavior probability model was developed:

(3) $$\begin{array}{l} \left\{ \begin{array}{l} p_{i}(a_{+})=\frac{e^{\left\{\varphi_{i0}+(\varphi_{i1}+\varphi_{i2}+\varphi_{i3})w_{i}\left(a_{+}\right)-(\varphi_{i4}+\varphi_{i5}+\varphi_{i6})w_{i}\left(a_{-}\right)\right\}}}{1+e^{\left\{\varphi_{i0}+(\varphi_{i1}+\varphi_{i2}+\varphi_{i3})w_{i}\left(a_{+}\right)-(\varphi_{i4}+\varphi_{i5}+\varphi_{i6})w_{i}\left(a_{-}\right)\right\}}}\\ p_{i}(a_{-})=1-p_{i}(a_{+})=1-\frac{e^{\left\{\varphi_{i0}+(\varphi_{i1}+\varphi_{i2}+\varphi_{i3})w_{i}\left(a_{+}\right)-(\varphi_{i4}+\varphi_{i5}+\varphi_{i6})w_{i}\left(a_{-}\right)\right\}}}{1+e^{\left\{\varphi_{i0}+(\varphi_{i1}+\varphi_{i2}+\varphi_{i3})w_{i}\left(a_{+}\right)-(\varphi_{i4}+\varphi_{i5}+\varphi_{i6})w_{i}\left(a_{-}\right)\right\}}}\\ =\frac{1}{1+e^{\left\{\varphi_{i0}+(\varphi_{i1}+\varphi_{i2}+\varphi_{i3})w_{i}\left(a_{+}\right)-(\varphi_{i4}+\varphi_{i5}+\varphi_{i6})w_{i}\left(a_{-}\right)\right\}}}\\ \frac{p_{i}(a_{+})}{p_{i}(a_{-})}=e^{\left\{\varphi_{i0}+(\varphi_{i1}+\varphi_{i2}+\varphi_{i3})w_{i}\left(a_{+}\right)-(\varphi_{i4}+\varphi_{i5}+\varphi_{i6})w_{i}\left(a_{-}\right)\right\}} \end{array}\right. \end{array}$$

where *φ_ij_* is the regression coefficient, *i* ∈ [1,2,…,N], *j* ∈ [1,2,…,6], and *φ_ij_* > 0. It should be noted that when *j* = 0, *φ_i_*_0_ ∈ (−∞,+∞). When *φ_i_*_0_ determines that the expected returns from the two different behavioral choices, i.e., proper and improper VA use, are both 0, that is, *w_i_* (*a*_+_) = *w_i_* (*a*_−_) = 0, the *i-*th actor’s behavior occurs without a driving force. The behavior probability in this case is called spontaneous probability. In fact, the farmer’s choice regarding VA use is influenced by their judgment of the expected return. Based on the behavior probability model, the probabilities of proper use *a*_+_ and improper use *a*_−_, *p_i_* (*a*_+_) and *p_i_* (*a*_−_), were simulated under the influence of farmers’ knowledge and return expectations. It is assumed that when *p_i_* (*a*_+_) ≥ *p_i_* (*a*_−_), the *i-*th actor chooses proper use; otherwise, they choose improper use. The group behavior probability was obtained by the observation of a total of *N* actors.

### 3.2. Simulation Experiment Description

In this study, the independence and interaction of individual pig farmers as an actor were simulated in a computer-generated environment when considering the influences of their knowledge and actor-to-actor information exchange on their VA use. The simulation was performed using Matlab based on Wu’s and Zhou’s research [22,24], and is described as follows:

(1) The simulation area is a 20 × 20 square area. At the start of the simulation, 100 farmers were randomly distributed in this area. Specific parameters are listed in Table 1 below.
(2) Vision values of farmers. Farmers’ VA use is closely related to the behavior of their peers [25]. “Vision value” was used to indicate the ability of farmers to collect surrounding information in the model. The larger the value, the higher the ability to collect surrounding information. At the start of the simulation, 100 vision values were randomly generated and assigned to each farmer. A vision value of two means that a farmer can observe the behaviors of other farmers in 2 × 4 grids surrounding them. It was assumed that: (a) If a farmer’s behavior is A, and the number of A within their range of vision ≥ the number of B, they will maintain their own behavior; otherwise, their behavior will change to B; and (b) if a farmer’s behavior is B, they will maintain their own behavior if the number of B within their range of vision ≥ the number of A; otherwise, their behavior will change to A.
(3) Knowledge of farmers. As set forth, *φ_i_*_1_ (the farmers’ knowledge of VA use specification), *φ_i_*_2_ (knowledge of hazards of VA residues), and *φ_i_*_3_ (knowledge of relevant laws and their penalties), take a value in [1,5] in the simulation, respectively, where 1 means no knowledge and 5 means complete knowledge. Based on the behavior probability model, *φ_i_*_1_, *φ_i_*_1_, and *φ_i_*_3_ are the coefficient part of proper use, and *φ_i_*_4_, *φ_i_*_5_, and *φ_i_*_6_ are the coefficient part of improper use. As proper and improper VA uses are two opposite behaviors, when a farmer has a high willingness to perform one behavior, the willingness to perform the other behavior will be relatively low. Therefore, it is assumed that the relationship between the two sets of coefficients is as follows:
  (4) $$\left\{ \begin{array}{l} \varphi_{i1}+\varphi_{i4}=5\\ \varphi_{i2}+\varphi_{i5}=5\\ \varphi_{i3}+\varphi_{i6}=5 \end{array}\right.$$
  To ensure scientific rigor and practical relevance of the simulation, the 100 farmers were assumed to have a lower-middle level of knowledge at the start of the simulation. It was assumed that *φ_i_*_0_ = 2 and *φ_i_*_1_ = *φ_i_*_2_ = *φ_i_*_3_ = 3, that is, the farmers’ three categories of knowledge fluctuated in the range of [1,3].
(4) Farmers’ expected return. The farmers’ expected return can be calculated by Equations (1) and (2). Farmers’ normal return, *G*, follows uniform distribution in [5,9] (in ten thousand yuan). *θ* is the ratio of farmers’ increased return from improper VA use to that from proper usage. In general, the higher the knowledge level regarding VA use specification, the lower the probability of an improper return. Therefore, *θ* is correlated with *φ_i_*_1_. To ensure that *θ* is nonnegative, it was assumed that *θ* + *φ_i_*_1_ = 5. Based on the finding of field interview regarding spot checks for pig farmers that were conducted by government regulators each year, the initial value of *q* was set to 0.3. According to the Regulations on Administration of Veterinary Drugs in China, the penalty for improper VA use was set to 30,000 yuan considering the various forms of improper use. Hence, *C*_1_ = 3. The higher the farming return, the higher the pressure from public opinion and moral pressure when the misconduct is disclosed and sanctioned. Hence, it is assumed that *C*_2_ = 2 × *G*.

## 4. Results and Discussion

### 4.1. Sample Characteristics

Statistical analysis was performed by SPSS 20.0 (SPSS Inc., Chicago, IL, USA). Demographic characteristics of respondents are shown in Table 2. According to our investigation, of the 654 pig farmers surveyed, 59.2% were male and 40.8% were female. The average age was 56.2 years. Generally, economic development in rural agricultural zones in China is lower than urban areas. The fact makes most younger generation workforce leave the rural agricultural zones to seek jobs in urban ones. This explains well why the average age of our sample is relatively high. Future studies can focus on the age factor and see if this variable can impact the relationship between knowledge and VA use. Furthermore, 58.7% of the respondents had an education level of primary school or below, and 28.4% had junior high school. A large proportion of respondents (51.4%) had a family size of five or more. The majority of respondents (66.1%) had a pig farming income accounting for 30% or less of total household income, and 78.9% of them had over ten years of pig farming experience.

### 4.2. Behaviors and Knowledge of Farmers Regarding Veterinary Antibiotics (VAs) Use

In terms of behaviors regarding VA use, 68.3% of the 654 pig farmers surveyed reported non-compliance with withdrawal time requirements, 55.5% overdosed VAs, and 24.3% used human antibiotics instead of VAs. Some farmers reported two or more types of improper VA use.

With regard to knowledge regarding VA use (Table 3), 78.0% and 19.3% of the respondents had no and little knowledge of VA use specification, respectively (97.3% altogether); 66.2% and 22.5% had no and little knowledge that antibiotics customized for humans cannot be used in pig farming (88.7% altogether); 48.2% and 28.9% had no and little knowledge of hazards of VA residues, respectively (77.1% altogether); and 64.7% and 22.0% had no and little knowledge of punishment for violating VA use regulations, respectively.

In terms of effect of government regulation, the majority of respondents (68.81%) believed that government supervision and inspection had no effect on their daily farming behaviors. Only 3.21% reported a great effect or a very great effect. Moreover, the vast majority of respondents (91.74%) were not penalized for violating VA use regulations. Only 7.34% and 0.92% were occasionally and frequently penalized for violations, respectively.

### 4.3. Simulation Experiment Results

The effects of each knowledge category on pig farmers’ VA use were simulated using Matlab. The effectiveness of government regulation on preventing and controlling pig farmers’ improper VA use was also analyzed. In figures regarding the simulation experiments, the black and gray curves indicate the probabilities of proper and improper uses in the farmer group, respectively.

#### 4.3.1. Influence of Knowledge about VA Use Specification on Farmer’s Behavioral Choices

**Figure 2:**
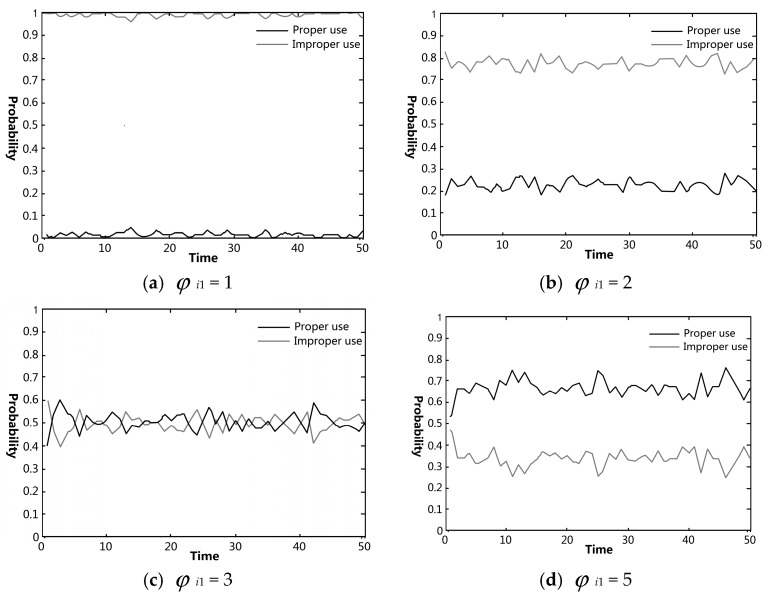
Simulation results of the changes of farmers’ behavioral choices regarding VA use under the variation of their knowledge about VA use specification: (**a**) the value of *φ_i_*_1_ (the farmers’ knowledge of VA use specification) was set to 1; (**b**) the value of *φ_i_*_1_ was set to 2; (**c**) the value of *φ_i_*_1_ was set to 3; (**d**) the value of *φ_i_*_1_ was set to 5.

The farmers were randomly distributed in the simulation area at the start of the simulation and then interacted with each other over time. Repeated experiments revealed relatively obvious curve changes when the value of knowledge of VA use specification, *φ_i_*_1_, was set to 1, 2, 3, and 5. Figure 2 reflects the co-variation between knowledge about VA use specification and behavioral choices of pig farmers. As shown in Figure 2, the behavioral choices of farmers appeared to have some regularity under the four different parameter settings of VA use knowledge of farmers—likelihood of good VA use behavior increases with increasing knowledge about VS use specification. When the value of *φ_i_*_1_ was 1, that is, the farmers generally have a low level of knowledge of VA use specification, there was a high probability of improper VA use, fluctuating between 95% and 100%, in the farmer group, as shown in Figure 2a. The probability of improper VA use decreased gradually when the value of *φ_i_*_1_ changed from 1 to 2 and 3. When *φ_i_*_1_ = 3, the probabilities of proper and improper uses fluctuated around 50%. When *φ_i_*_1_ further increased to 5, the probability of improper VA use was significantly lower than that of proper use. The above findings indicated that the probability of improper VA use decreased with an increase in the farmers’ knowledge of VA use specification. This was consistent with the conclusion of Wu [26]. However, the probability of improper VA use was still higher than that of proper use. Only when the level of knowledge was sufficiently high were farmers inclined to use VAs properly. Also, such result echoes to the finding of Pham and colleagues [27] that the farmers seldom know the real and specific purpose of using VA. Therefore, persistent improvement of pig farmers’ knowledge about VA use specification plays a fundamental role in promoting proper VA use. Note that in the model, the x-axis represents a parameter of time, but we did not specifically assign a time unit for that parameter. By not specifying time period can extend the flexibility and generalizability of the models and results [24].

#### 4.3.2. Influence of Knowledge about the Hazards of VA Residues on Farmer’s Behavioral Choices

**Figure 3:**
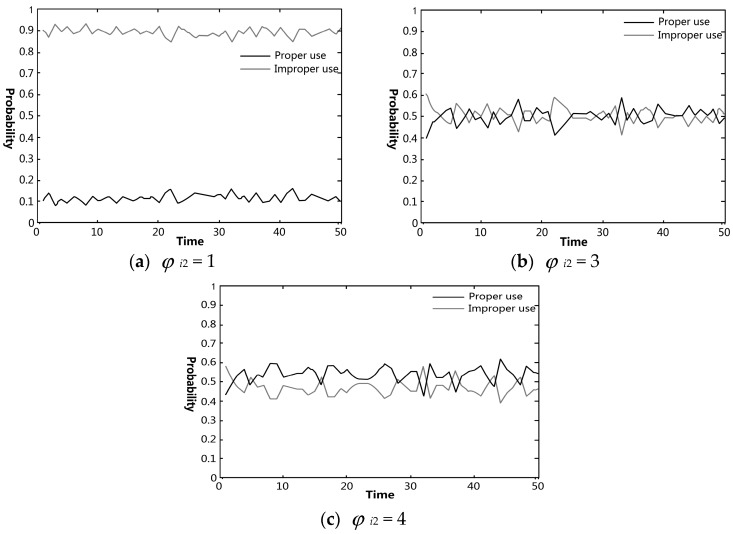
Simulation results of the changes of farmers’ behavioral choices regarding VA use under the variation of their knowledge about hazards of VA residues: (**a**) the value of *φ_i_*_2_ (knowledge of hazards of VA residues) was set to 1; (**b**) the value of *φ_i_*_2_ was set to 3; (**c**) the value of *φ_i_*_2_ was set to 4.

Figure 3 demonstrates the relationship between farmers’ knowledge about the hazards of VA residues on the behavioral choices of them. As can be seen from Figure 3a (*φ_i_*_2_ = 1), when farmers had no knowledge about the hazards of VA residues, there was a high probability of improper VA use, fluctuating around 90%, in the farmer group. This result was consistent with the survey finding that respondents with improper VA use had a poor knowledge about the hazards of VA residues. Moreover, the probability of proper VA use increased significantly when the whole group’s knowledge about the hazards of VA residues increased to a certain level, as shown in Figure 3b. When *φ_i_*_2_ = 3, the probabilities of improper and proper VA use fluctuated between 40% and 60%. A comparison of Figure 3b (*φ_i_*_2_ = 3) and 3c (*φ_i_*_2_ = 4) indicated that the probability of improper VA use did not significantly decrease with the further increase in knowledge about the hazards of VA residues. One possible reason is the difficulties in government regulation due to decentralized farming. Moreover, the economic benefits from improper VA use in pig farming are attractive enough for most farmers due to the general absence of strict supervision and punishment by the government [26]. Therefore, it is necessary to educate farmers about the hazards of improper VA use, and at the same time impose financial penalties for improper VA use to reduce willful misconduct.

#### 4.3.3. Influence of Knowledge about the Relevant Laws and Their Penalties on Farmer’s Behavioral Choices

**Figure 4:**
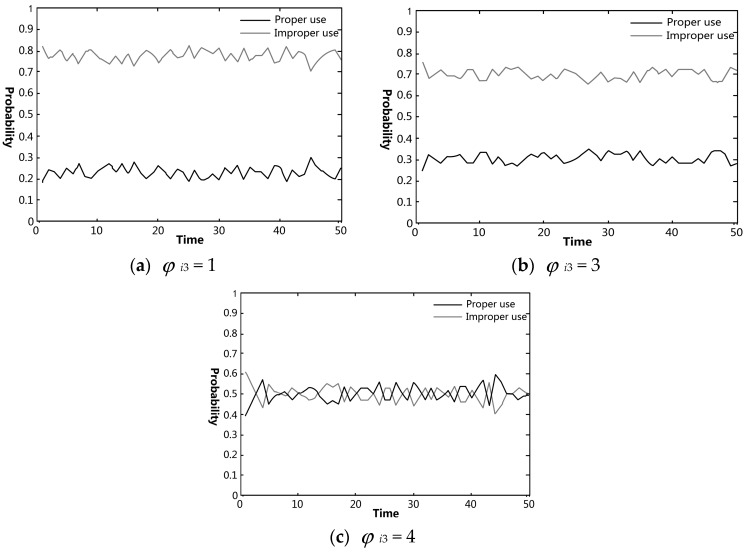
Simulation results of the changes of farmers’ behavioral choices regarding VA use under the variation of their knowledge about relevant laws and their penalties: (**a**) the value of *φ_i_*_3_ (knowledge of relevant laws and their penalties) was set to 1; (**b**) the value of *φ_i_*_3_ was set to 3; (**c**) the value of *φ_i_*_3_ was set to 4.

Figure 4 demonstrates the influences of knowledge about the relevant laws and penalties on farmers’ behavioral choice. Farmers’ VA use is closely related to their knowledge about relevant laws and their penalties. When farmers’ knowledge about relevant laws and their penalties *φ_i_*_3_ = 1, there was a relatively high probability of improper VA use, fluctuating between 70% and 80%, as shown in Figure 4a,b. When the value of *φ_i_*_3_ changed from 1 to 3, the probability of proper VA use in the farmer group did not increase substantially, while the probability of improper use decreased by 5–10%. As can be seen from Figure 4c, when farmers had a relatively high level of knowledge about the relevant laws and their penalties, improper VA use still occurred at a probability of around 50%, which was similar to the probability of proper use. In fact, current pre- and post-slaughter pig quarantine in China only focuses on foot-and-mouth disease, swine fever, swine vesicular disease, and other diseases. VA residues in live pigs are not strictly monitored. The testing of antibiotic residues only includes several common types of VAs. This has resulted in a low probability of discovering improper VA use by farmers, and consequently, there has been insufficient punishment. From the perspective of policy regulation, pig farmers in China is allowed to execute routine treatments by themselves, just like some advanced nations including The Netherlands (e.g., Kuipers et al., 2016 [21]). This and other similar permissions have allowed farmers in China more autonomy in medical related behaviors. Therefore, it is possible that farmers, driven by economic interests and endorsed with higher behavioral autonomy, deliberately choose improper VA use, despite knowing the penalties.

### 4.4. Influence of Government Regulation on Farmer’s Behavioral Choices

**Figure 5:**
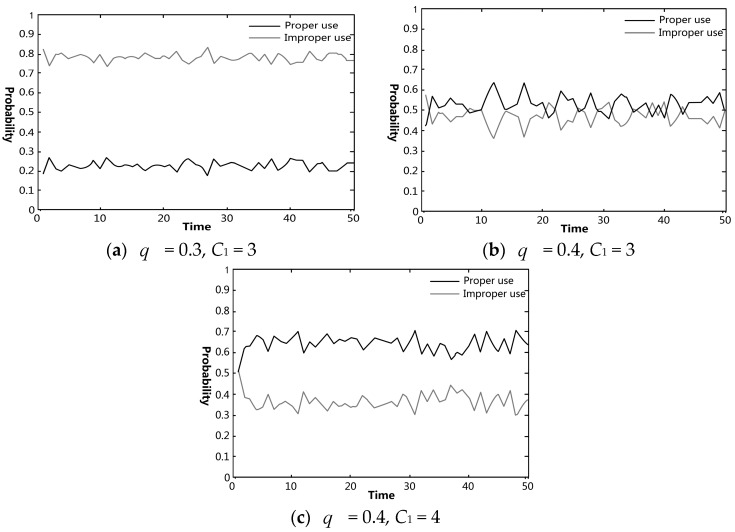
Simulation results of the changes of farmers’ behavioral choices regarding VA use under the variation of government regulation: (**a**) the probability of the farmers’ improper VA use checked by government regulators was set to 0.3 (*q* = 0.3), and the penalty for improper VA use was set to 30,000 yuan (*C*_1_ = 3); (**b**) *q* = 0.4, *C*_1_ = 3; (**c**) *q* = 0.4, *C*_1_ = 4.

Regulative tactics can influence antibiotic use in different ways [28]. Hence, in our simulation experiments, government regulation of pig farmers’ VA use was reflected by spot checks and penalties for improper use. The experimental results are shown in Figure 5, which illustrates the relationships between government regulation (in terms of different numbers of random checking and amount of penalty) and farmers’ VAs use. When the sampling rate in spot checks and the penalties were both low, the proportion of farmers with improper VA use (approximately 80%) was much larger than that with proper use (approximately 20%). When the sampling rate in spot checks increased, the proportion of farmers with proper VA use (fluctuating between 50% and 60%) was slightly higher than that with improper use. Furthermore, when the penalties were increased, the number of farmers with proper VA use was significantly higher than that with improper use. This was consistent with the findings of Chen et al. [29] on the behaviors of pig farmers.

## 5. Conclusions and Policy Implications

In this study, the dynamic changes in farmers’ behavioral choice regarding VA use were observed by simulation when considering their knowledge regarding VAs and farmer-to-farmer interaction. First, the simulation results showed that the probability of improper VA use decreased with the increase in farmers’ knowledge about VA use specification. When the level of this knowledge was high enough, farmers were inclined to make proper use of VAs. In short, their use of VAs was significantly affected by their knowledge about VA use specification. Second, the probability of improper VA use decreased at a decreasing rate as farmers’ knowledge about the hazards of VA residues increased. In general, the farmers’ use of VAs is related to their knowledge about the hazards of VA residues. Third, when farmers had a high level of knowledge about the relevant laws and their penalties, there was still a high probability of improper VA use, which was similar to that of proper use. The farmers’ choice regarding the use of VAs was not significantly affected by their knowledge about the relevant laws and their penalties.

These important findings call for the improvement of VA management policies and the development of sustainable interventions in China to prevent the improper use of VAs by pig farmers, in order to reduce antibiotic resistance and improve pork safety for the protection of public health. Considering the fact that improper VA use is common among pig farmers in China, the following policy recommendations are offered based on the above conclusions. First, support should be provided to help farmers, the end-user of VAs, to improve their knowledge about VA use specification and to keep records of VA use. Changes of management practices (e.g., veterinary professionals’ involvement and professionals-farmers communications) may help increase the level of farmers’ awareness [21]. Second, support should be provided to help farmers understand the hazards of VAs and thus make proper use of them. Nonetheless, such provision of supportive resources and information should be highly relevant to farmers’ special situations (e.g., Garforth et al., 2013 [30]), so as to be highly appreciated and adopted by farmers. For farmers with different levels of knowledge, specific and different resources and information should be endorsed in different ways. For example, for farmers with lower knowledge level of VA, more visual (non-text) and life-related case stories should be told, while for farmers with higher-level knowledge, more systematic information and resource packages should be supplied. Third, as the food safety regulator, the government should improve and publicize relevant laws and regulations to enhance the legal awareness of farmers [28]. Moreover, the government should enhance supervision and inspection, increase the sampling rate in spot checks, and impose harsher penalties for improper VA use.

## Figures and Tables

**Table 1:** Settings of experimental parameters.

Model Parameters	Parameter Value (Symbol)
Area	20 × 20
Total number of farmers, *N*	100
Farmers: proper use	A
Farmers: improper use	B
Vacancy	O

**Table 2:** Demographic characteristics of the surveyed farmers.

Characteristics	Categories	Frequency (*n*)	Percentage (%)
Gender	Male	387	59.2
	Female	267	40.8
Education Attainment	Primary school and lower	384	58.7
	Middle school	186	28.4
	High School and Above	84	12.9
Number of household members	1	12	1.8
	2	57	8.7
	3	93	14.2
	4	156	23.9
	5 or more	336	51.4
Proportion of pig production to family income	30% or less	432	66.1
	31–50%	78	11.9
	51–80%	54	8.3
	81–90%	33	5.0
	91% or more	57	8.7
Years of farming	1–3 years	45	6.9
	4–6 years	42	6.4
	7–10 years	51	7.8
	Over 10 years	516	78.9
Slaughter amount	1–30 pigs	417	63.8
	31–100 pigs	135	20.6
	Over 100 pigs	102	15.6

**Table 3:** Farmers’ knowledge about VAs (in %).

Knowledge	1 = No Knowledge	2 = Little Knowledge	3 = Moderate Knowledge	4 = Good Knowledge	5 = Complete Knowledge
VAs should be used as directed by a veterinarian in strict accordance with the manufacturer’s instructions	78.03	19.27	0.30	1.22	1.22
Antibiotics customized for human cannot be used in pig farming	66.21	22.48	1.22	7.34	2.75
VA residues can cause antibiotic resistance and endanger human health	48.17	28.90	7.80	12.84	2.29
Farmers will be punished by the government for improper VA use	64.68	22.02	3.67	8.26	1.37
